# Interference of urinary albumin-to-creatinine ratio measurement by glycosuria: clinical implications when using SGLT-2 inhibitors

**DOI:** 10.1016/j.kint.2022.12.027

**Published:** 2023-02-02

**Authors:** Daniel Chapman, Parminder K. Judge, Rebecca J. Sardell, Natalie Staplin, Thomas Arnold, Doreen Zhu, Sarah Ng, Stewart Moffat, Martin J. Landray, Colin Baigent, Michael Hill, Richard Haynes, Sarah Clark, William G. Herrington

**Affiliations:** 1 Clinical Trial Service Unit and Epidemiological Studies Unit, Nuffield Department of Population Health, University of Oxford, Oxford, UK; 2 Medical Research Council–Population Health Research Unit, Nuffield Department of Population Health, University of Oxford, Oxford, UK

**Keywords:** glycosuria, interference, sodium glucose cotransporter-2 in-hibitor, urine albumin-to-creatinine ratio

Urinary albumin-to-creatinine ratio (uACR) measured on spot samples provides a convenient method to screen for, diagnose, stage, and monitor chronic kidney disease (CKD),^1^ and change in albuminuria is proposed as a surrogate outcome for CKD progression in clinical trials.^2^ Sodium glucose cotransporter-2 (SGLT-2) inhibitors are increasingly used because of important effects on risk of cardiovascular disease and CKD progression.^3^ Under normoglycemic and modest hyperglycemic conditions, SGLT-2 inhibitors increase urinary glucose excretion to ~50–80 g/d, and to >100 g/d in people with diabetes and glomerular hyperfiltration. This can equate to urinary glucose concentrations up to ~500 mmol/L.

High serum glucose is known to interfere with the Jaffe reaction used to measure serum creatinine, with bias exceeding 10% with glucose concentrations >20 mmol/L in some assays.^4^ This problem can be circumvented by use of enzymatic creatinine assays,^4,5^ but such assays are more expensive and use is often low, even in high-income countries (only ~50% of UK clinical laboratories use enzymatic methods).^6^ Like in serum, glucose may interfere with urinary creatinine measurement, and the potentially high levels of glycosuria resulting from SGLT-2 inhibitor use could cause biases that have important clinical implications.^7^ We aimed to quantify this potential bias using laboratory interference studies in urine samples from patients with CKD spiked with a range of glucose concentrations intended to represent the range expected in patients taking SGLT-2 inhibitors.

## Results

Laboratory and statistical methods are provided in the Supplement (with assay repeatability measurements in Supplementary Table S1). We studied urine samples from 370 patients with CKD: 37 (10%) samples with endogenous glycosuria ≥5.6 mmol/L were excluded, leaving 333 participants’ samples for analyses. Median (interquartile range) uACR was 63 (17–150) mg/mmol, with 30 (9%), 72 (22%), and 231 (69%) with uACRs of <3, ≥3 to <30, and ≥30 mg/mmol, respectively (Supplementary Table S2). Median (interquartile range) urinary glucose concentration before spiking with glucose was 0.33 (0.33–0.57) mmol/L.

### Interference studies assessed using log uACR

**Figure 1:**
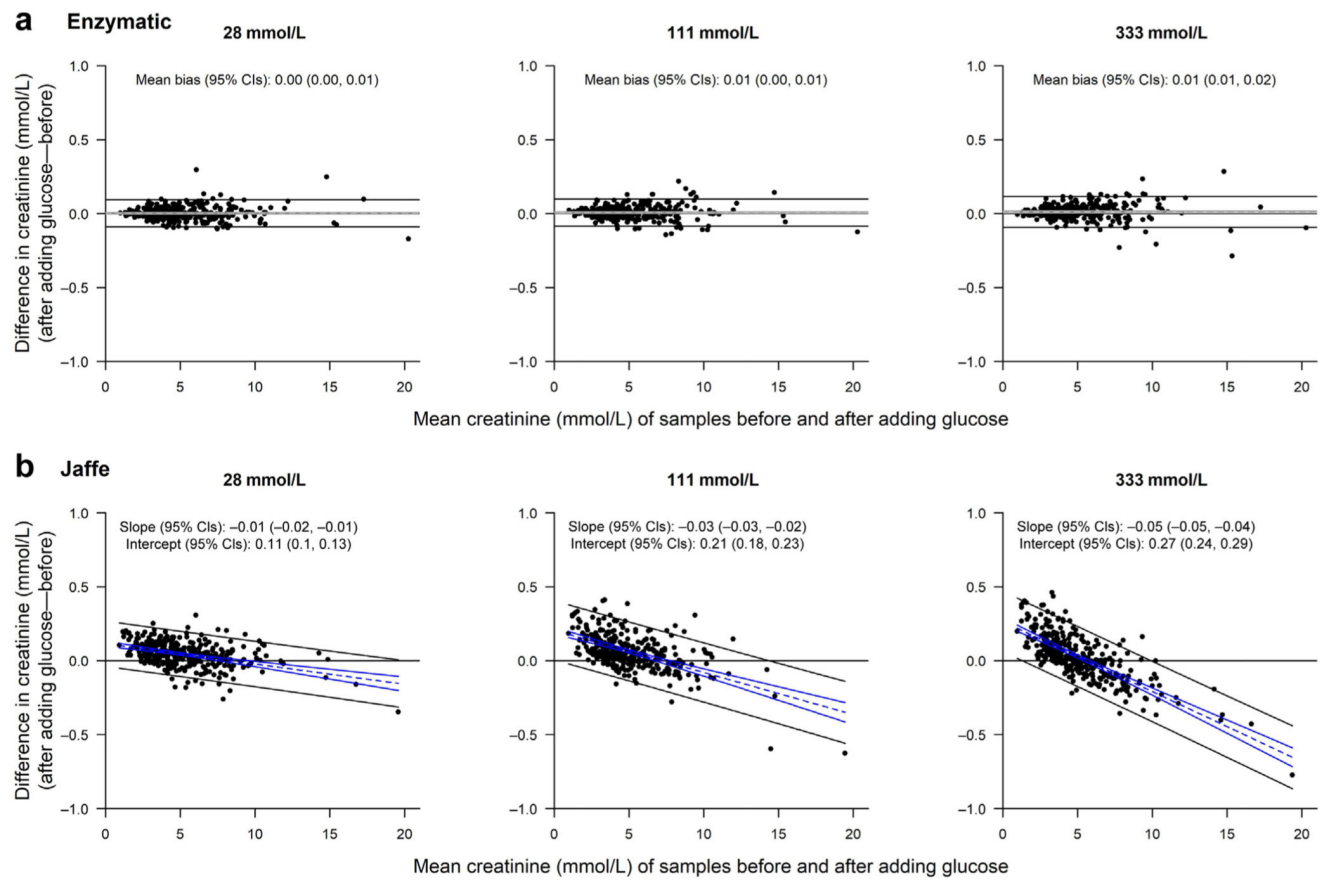
Bland-Altman plots for creatinine, by glucose concentration. Mean difference and 95% confidence intervals (CIs) are shown by dashed and solid gray lines. If slope is significantly different from 0, bias is instead shown by a regression line in blue. The 95% limits of agreement are shown as solid black lines.

**Figure 2:**
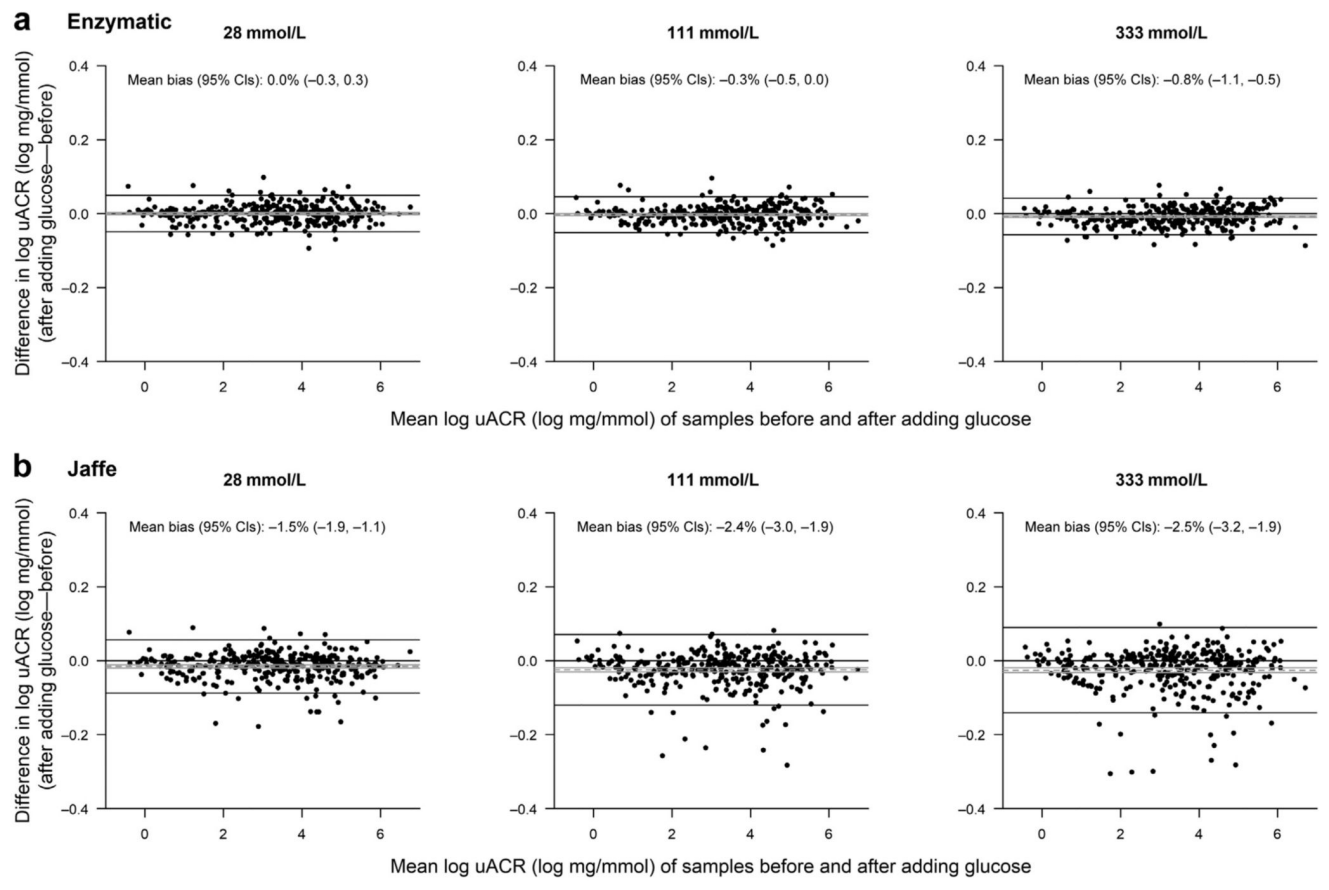
Bland-Altman plots for urinary albumin-to-creatinine ratio (uACR), by glucose concentration. Mean bias and 95% confidence intervals (CIs) are shown by dashed and solid gray lines. The 95% limits of agreement are shown as solid black lines. For log-transformed variables, mean bias values have been back-transformed onto the original scale to give a percentage difference.

There was no evidence that spiking with glucose had any effect on urinary albumin measurements at 28 or 111 mmol/L glucose concentration, but a 0.5% bias emerged at 333 mmol/L (Supplementary Figure S1). There was no bias for enzymatic creatinine measurements (Figure 1a). Consequently, overall, there was only a small bias in log uACR measurement when an enzymatic method was used and when urine glucose concentration was 333 mmol/L (Figure 2a).

For the Jaffe creatinine method, however, the presence of glucose caused a bias that resulted in substantial over-estimation of urinary creatinine at the lowest creatinine concentrations and a small underestimate at high urinary creatinine concentrations (Figure 1b). This bias was not importantly different across the range of levels of albuminuria (Supplementary Figure S2). The net bias resulting from glucose interference was, on average, to underestimate uACR across the range of albumiuria studied. The Bland-Altman plots in Figure 1b show increasingly steep regression line slopes with higher glucose concentration, indicating increasing bias with higher glucose concentrations. In this cohort, the presence of 28 mmol/L of glucose in the urine resulted in a –1.5% mean bias in uACR (95% confidence interval, –1.9% to –1.1%), which increased to –2.5% (95% confidence interval, –3.2% to –1.9%) at a glucose concentration of 333 mmol/L (Figure 2b). Bias was largest at low creatinine concentrations (i.e., in dilute urine; Supplementary Figure S3). Scatterplots of paired creatinine and uACR measurements are provided in Supplementary Figures S4 and S5.

### Illustrations of the impact of glucose interference on uACR (original scale)

In this particular CKD cohort, where taking SGLT-2 inhibitors was simulated with glucose spiking, interference from the highest level of glucose concentration led to 5.1% (17/333) and 1.8% (6/333) of samples having uACR underestimates of ≥10% and ≥20%, respectively (Supplementary Table S3). We also estimated absolute and percentage change in uACR for different hypothetical levels of uACR. Among the 4% (14/333) of participants with a urine creatinine <2.5 mmol/L, the presence of 28 mmol/L of urinary glucose caused a bias of –5.2% to –7.0% (depending on level of uACR). This bias increased to –10.1% to –13.0% at a concentration of 333 mmol/L. In comparison, biases were all <1.0% for those with urinary creatinine of ≥5 mmol/L (Supplementary Table S4). Results using untransformed values of albumin and uACR are provided in Supplementary Figures S6 and S7.

## Discussion

Jaffe assays are commonly used to measure creatinine, and the presence of glycosuria in the range expected to result from use of SGLT-2 inhibitors causes a biased underestimate of uACR when such an assay is used. This bias increases progressively with higher urinary glucose concentrations, and particularly affects dilute urine samples (i.e., urinary creatinine <2.5 mmol/L).^5^ Any underestimation of uACR by glucose interference creates a positive bias for any observed reduction in uACR in serial uACR measurements. In patients with CKD stages 3 to 4 in this study, high urinary glucose concentration resulted in an ~10% underestimate of uACR among those with dilute urine. In contrast, enzymatic methods were almost unaffected. Such a level of bias is arguably unacceptable if it alters decisions made by clinicians unaware of the interference. An overestimate of the reduction in albuminuria observed after starting an SGLT-2 inhibitor could, for example, result in a decision not to start other proven kidney disease–modifying treatments or make a patient ineligible for a treatment reserved for people with a certain level of albuminuria.

This bias also has implications for analyses from historical and design of future clinical trials. A 30% reduction in geometric mean uACR has been suggested as a meaningful and valid surrogate of treatments for progressive CKD.^8^ Although urinary glucose resulting from SGLT-2 inhibition is unlikely to interfere with Jaffe assays sufficiently to result in a ≥30% change, it could result in overestimated or misleading claims of beneficial effects on uACR. In a review of the published literature, we were only able to identify type of urine assay used in 1 of 11 large placebo-controlled SGLT-2 inhibitor trials and none of 4 intensive versus standard glycemic control trials (Supplementary Table S5).^9^

Given the increasing use of SGLT-2 inhibitors in clinical practice, we suggest uACR measured using Jaffe creatinine assays should be avoided, where possible. Alternatively, where resource limitations preclude use of more expensive enzymatic methods, assay type should be reported with results. Methods traceable to the international standard not affected by glycosuria should be used in trials assessing effects of interventions on albuminuria when such interventions could modify glycosuria.

## Supplementary Material

Supplementary Materials

## Data Availability

Data used in this publication are available in line with the policy and procedures described at: https://www.ndph.ox.ac.uk/data-access. For further information, contact the corresponding author.
