# Supplementary material for: Interference of urinary albumin-to-creatinine ratio measurement by glycosuria: clinical implications when using SGLT-2 inhibitors
Source: Kidney Int. Author manuscript; Available in PMC 2025 Jul 4. (PMC7617837; doi:10.1016/j.kint.2022.12.027)
Supplement: Supplementary Materials [file EMS206645-supplement-Supplementary_Materials.docx]

**Supplementary Material**

**Interference of urinary albumin-to-creatinine ratio measurement by glycosuria: clinical implications when using SGLT-2 inhibitors**

Contents

[**Supplementary Methods** 2](#_Toc121917082)

[**Supplementary Tables** 4](#_Toc121917083)

[Table S1: Assay repeatability coefficient of variation determined from measuring quality control material over 20 days, with two runs per day and two replicates per run. 4](#_Toc121917084)

[Table S2: Baseline characteristics 5](#_Toc121917085)

[Table S3: Percentage of samples with reduction in uACR due to glucose spiking 6](#_Toc121917086)

[Table S4: Absolute and percent change in uACR due to glucose interference of urine Jaffe assays by hypothetical levels of uACR 7](#_Toc121917087)

[Table S5: Methods of albuminuria measurement in other clinical trials 8](#_Toc121917088)

[**Supplementary Figures** 9](#_Toc121917089)

[Figure S1: Bland-Altman plots for albumin, by glucose concentration 9](#_Toc121917090)

[Figure S2: Association between creatinine and the difference in uACR before and after adding glucose to a Jaffe assay, by glucose level and uACR at randomization 10](#_Toc121917091)

[Figure S3: Association between mean creatinine and the difference in uACR before and after adding glucose, by assay method and glucose concentration 11](#_Toc121917092)

[Figure S4: Creatinine before and after adding glucose, by glucose concentration 12](#_Toc121917093)

[Figure S5: uACR before and after adding glucose, by glucose concentration 13](#_Toc121917094)

[Figure S6: Bland-Altman plots for uACR (untransformed), by glucose concentration 14](#_Toc121917095)

[Figure S7: Bland-Altman plots for albumin (untransformed), by glucose concentration 16](#_Toc121917096)

[**Supplementary References** 17](#_Toc121917097)

# **Supplementary Methods**

Early morning urine samples collected and stored as part of the UK Heart and Renal Protection (HARP)-III trial (previously reported)^S1^ were used for laboratory interference experiments. UK HARP-III randomized 414 participants ≥18 years with CKD stages 3/4 to irbesartan or sacubitril/valsartan. Urine samples have been stored at -80^o^C, a temperature reported to maintain stability of uACR measurement, prior to analysis.^S2,S3^ 370 participants’ samples with a urine volume of at least 1mL were available for this study. The UK HARP-III trial received ethical approval from Nottingham Research Ethics Committee 2 [13/EM/0434]) and regulatory approvals before the enrollment of any study participants.

*Laboratory methods*

Each urine sample was thawed, mixed by inversion, and samples separated into four 245µl aliquots. One aliquot had 70µl of deionised water added (reference sample), the remaining aliquots were spiked with 70µl of either 125, 500 or 1500 mmol/L glucose solution (produced by diluting Merck product: G8769 in deionised water). This provided a final glucose concentration of 28, 111 and 333 mmol/L, respectively (the range expected in patients on SGLT-2 inhibitors or with poorly controlled diabetes).^S4^ All assay measurements were made on a Beckman Coulter DxC700AU Clinical Chemistry Analyser (Beckman Coulter, Inc., Brea CA) using the manufacturer’s commercially available kits and protocols. Urine albumin was measured by immunoturbimetric method traceable to IFCC Standard CRM470 (product number B38858), urine creatinine was measured using both Jaffe and enzymatic methods traceable to NIST SRM 3667 (product numbers OSR6178 and OSR61204, respectively), and urinary glucose was measured using enzymatic method (hexokinase) traceable to NIST 965b L4 (product number OSR6121). The NDPH Wolfson is a UKAS accredited testing laboratory No 2799 and these assays were on their ISO/IEC 17025 Schedule of Accreditation at the time of this study. Assay repeatability and within laboratory precision were assessed using protocols based on the Clinical & Laboratory Standards Institute (CLSI) guideline EP05 Evaluation of Precision of Quantitative Measurement Procedures, 3rd Edition. Reference and spiked samples were measured in the same run to reduce analytical variability. Assay repeatability coefficient of variation are summarized in Table S1. Our albumin assay has been shown to be biased compared to LC-MS/MS by between -9 to -20% at urine albumin median concentrations by of 16 to 184 mg/L.^S5^

*Statistical analyses*

Analyses used Bland Altman plots comparing the mean and difference of paired albumin, creatinine and uACR measurements. Reference and spiked samples were obtained for each level of glucose spiking.^S6^ For assessments of bias, albumin and uACR (variables not normally distributed),^S7,S8^ measurements were log-transformed prior to plotting. Creatinine values were normally distributed, therefore log transformation was not required. Results using untransformed values of albumin and uACR are also provided in Supplementary Figures S6 and S7. Where the slope between the mean and difference of the samples was significantly different from zero (i.e. the bias between reference and spiked samples was not constant), a regression line was fitted. The difference in log uACR before and after the addition of glucose was also plotted against mean creatinine, both overall and by subgroups of uACR at randomization. For samples with uACR below the measureable range, the lower limit of quantification was imputed. Additionally, scatterplots of paired creatinine and uACR measurements (Figures S4 and S5 respectively) are presented, with Lin’s concordance correlation coefficient used to assess the degree of agreement between the two measurements. Analyses were performed using SAS version 9.4 and R version 4.1.2.

# **Supplementary Tables**

## Table S1: Assay repeatability coefficient of variation determined from measuring quality control material over 20 days, with two runs per day and two replicates per run.

| **Urine assay** | **Mean Concentration** | **Units** | **Standard Deviation** | **Coefficient of variation (%)** |
| --- | --- | --- | --- | --- |
| Albumin | 15.87 | mg/L | 0.195 | 1.23 |
|  | 144.90 | mg/L | 2.065 | 1.43 |
| Jaffe Creatinine | 5.78 | mmol/L | 0.059 | 1.03 |
|  | 12.33 | mmol/L | 0.114 | 0.92 |
| Enzymatic Creatinine | 6.35 | mmol/L | 0.027 | 0.42 |
|  | 12.31 | mmol/L | 0.059 | 0.48 |
| Glucose | 2.09 | mmol/L | 0.016 | 0.79 |
|  | 19.70 | mmol/L | 0.090 | 0.46 |

## Table S2: Baseline characteristics

| **Variable** | **n=333** |  |  |
| --- | --- | --- | --- |
| **Age** |  |  |  |
| Mean (SD) | 62 (14) |  |  |
| <50y | 63 (19%) |  |  |
| ≥50y-<70y | 158 (47%) |  |  |
| ≥70y | 112 (34%) |  |  |
| **Sex** |  |  |  |
| Male | 245 (74%) |  |  |
| Female | 88 (26%) |  |  |
| **Ethnicity** |  |  |  |
| White | 303 (91%) |  |  |
| South Asian | 14 (4%) |  |  |
| Black | 7 (2%) |  |  |
| Other | 9 (3%) |  |  |
| **Prior diabetes** | 107 (32%) |  |  |
| **CKD-EPI estimated glomerular filtration rate at randomisation (mL/min/1.73m²)** |  |  |  |
| Mean (SD) | 35.4 (10.8) |  |  |
| <30 | 126 (38%) |  |  |
| ≥30 to <45 | 140 (42%) |  |  |
| ≥45 | 66 (20%) |  |  |
| Not available | 1 |  |  |
| **Urine albumin-to-creatinine ratio at randomisation (mg/mmol)** |  |  |  |
| Geometric mean (approximate SE) | 43 (4) |  |  |
| Median (IQR) | 63 (17-150) |  |  |
| <3 | 30 (9%) |  |  |
| ≥3 to <30 | 72 (22%) |  |  |
| ≥30 | 231 (69%) |  |  |
| **Urine creatinine at randomisation (mmol/L)** |  |  |  |
| Geometric mean (approximate SE) | 6.06 (0.16) |  |  |
| Median (IQR) | 6.18 (4.20-8.34) |  |  |
| <2.5 | 14 (4%) |  |  |
| ≥2.5 to <5 | 105 (32%) |  |  |
| ≥5 | 214 (64%) |  |  |
| **Glucose concentration before spiking (mmol/L)** |  |  |  |
| Median (IQR) | 0.33 (0.33-0.57) |  |  |
| Values are n (%), mean (SD), geometric mean (˜SE), or median (IQR).  CKD-EPI indicates Chronic Kidney Disease Epidemiology Collaboration; IQR, interquartile range.  No participants were taking a sodium glucose cotransporter-2 inhibitor. | | | |

## Table S3: Percentage of samples with reduction in uACR due to glucose spiking

| **Assay** | **Glucose concentration (mmol/L)** | **n (%) of samples with reduction in uACR due to glucose spiking alone** | | |
| --- | --- | --- | --- | --- |
|  |  | **≥10%** | **≥20%** | **≥30%** |
| Enzymatic | 28 | 0 | 0 | 0 |
|  | 111 | 0 | 0 | 0 |
|  | 333 | 0 | 0 | 0 |
| Jaffe | 28 | 7 (2%) | 0 | 0 |
|  | 111 | 12 (4%) | 4 (1%) | 0 |
|  | 333 | 17 (5%) | 6 (2%) | 0 |

uACR=urinary albumin-to-creatinine ratio, which is presented on its original scale.

| Table S4: Absolute and percent change in uACR due to glucose interference of urine Jaffe assays by hypothetical levels of uACR | | | | | | |  |
| --- | --- | --- | --- | --- | --- | --- | --- |
|  |  |  |  |  |  |  |  |
| **Glucose concentration (mmol/L)** | **Level of uACR (mg/mmol)** | **Urine creatinine concentration** | | | | | |
|  |  | **<2.5 mmol/L** | | **≥2.5 to <5 mmol/L** | | **≥5 mmol/L** | |
|  |  | **Absolute change (mg/mmol)** | **%** | **Absolute change (mg/mmol)** | **%** | **Absolute change (mg/mmol)** | **%** |
| 28 | 3 | -0.21 | -7.0% | -0.01 | -0.3% | -0.02 | -0.7% |
| 28 | 30 | -1.55 | -5.2% | -0.48 | -1.6% | 0.04 | 0.1% |
| 28 | 300 | -17.39 | -5.8% | -6.29 | -2.1% | -0.99 | -0.3% |
| 111 | 3 | -0.28 | -9.3% | -0.05 | -1.7% | 0.02 | 0.7% |
| 111 | 30 | -2.86 | -9.5% | -0.84 | -2.8% | 0.06 | 0.2% |
| 111 | 300 | -31.22 | -10.4% | -9.61 | -3.2% | -0.24 | -0.1% |
| 333 | 3 | -0.32 | -10.7% | -0.03 | -1.0% | 0.01 | 0.3% |
| 333 | 30 | -3.04 | -10.1% | -0.92 | -3.1% | 0.23 | 0.8% |
| 333 | 300 | -39.11 | -13.0% | -11.61 | -3.9% | 2.71 | 0.9% |
| uACR=urinary albumin-to-creatinine ratio, which is presented on its original scale.  Percentage change in uACR is calculated as e^b^-1, where b is the mean absolute bias in log uACR for the stated glucose concentration and absolute change in uACR is calculated by this proportional change to the hypothetical level of uACR. Mean creatinine concentration in the <2.5, ≥2.5 to <5 mmol/L, and ≥5 mmol/L groups were 1.8, 3.7 and 7.4 mmol/L respectively. | | | | | | | |

## Table S5: Methods of albuminuria measurement in other clinical trials

| **Trial acronym** | **Urinary albumin-to-creatinine ratio (uACR)**  **measurement method (as recorded)** |
| --- | --- |
| **SGLT2 inhibitor trials** | |
| DECLARE-TIMI 58^S9,S10^ | Unclear |
| CANVAS Program^S11,S12^ | uACR was measured every 26 weeks in CANVAS-R, and at week 12 and then annually in CANVAS. Serum creatinine measurement with estimation of GFR was undertaken in a central laboratory using the Jaffe method with rate blanking |
| VERTIS CV^S13^ | Unclear |
| EMPA-REG OUTCOME^S14,S15^ | Unclear |
| DAPA-HF^S16^ | Unclear |
| EMPEROR-REDUCED^S17^ | Unclear |
| EMPEROR-PRESERVED^S18^ | Unclear |
| CREDENCE^S11^ | Unclear |
| SOLOIST-WHF^S19^ | Not tested |
| SCORED^S20^ | Unclear. |
| DAPA-CKD^S21^ | Unclear. |
| **Other trials** | |
| ACCORD^S22^ | Unclear |
| UKPDS^S23,S24^ | Unclear (from 1988, urine albumin measured by immunoturbidimetric method) |
| ADVANCE^S25^ | Unclear |
| VADT^S26^ | Unclear |

# **Supplementary Figures**

## Figure S1: Bland-Altman plots for albumin, by glucose concentration


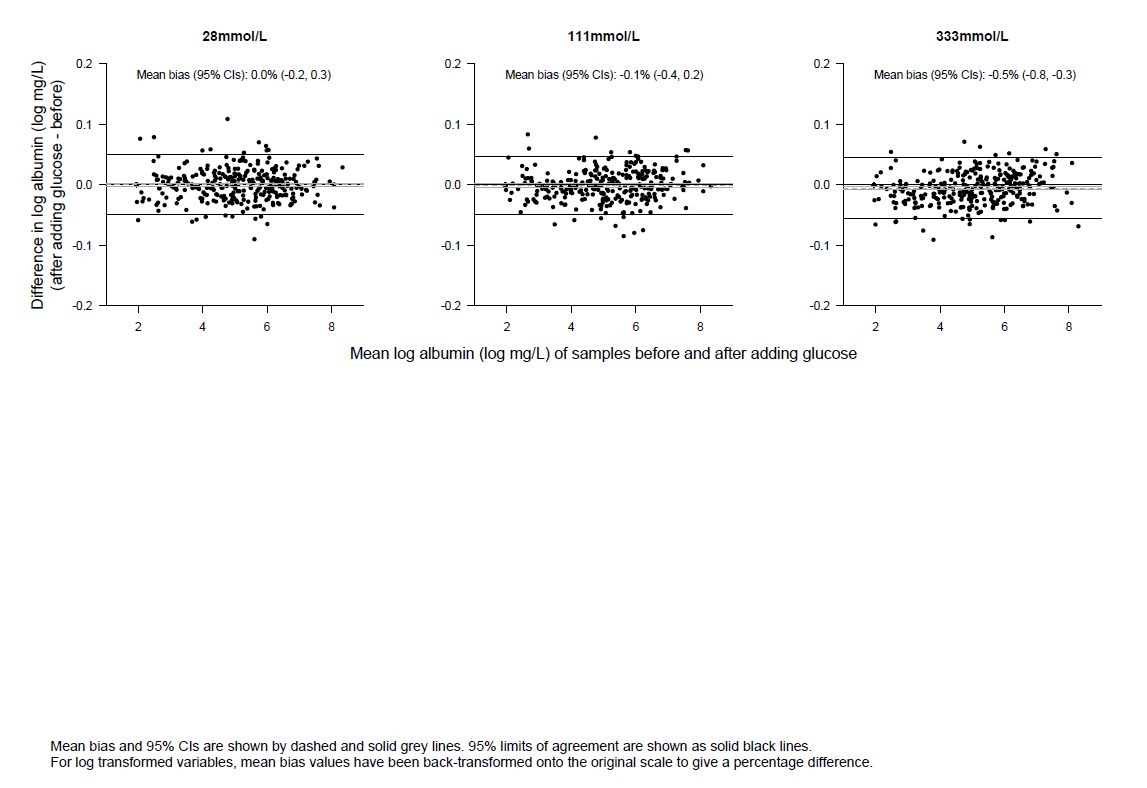


## Figure S2: Association between creatinine and the difference in uACR before and after adding glucose to a Jaffe assay, by glucose level and uACR at randomization


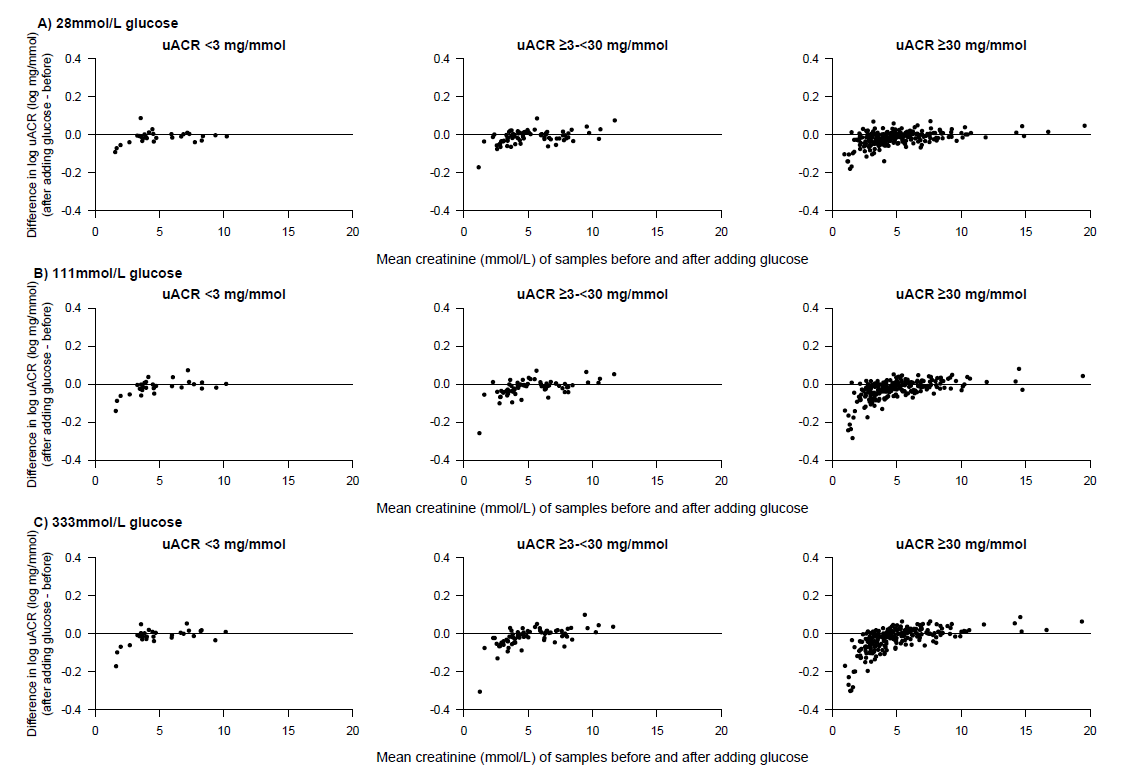


## Figure S3: Association between mean creatinine and the difference in uACR before and after adding glucose, by assay method and glucose concentration


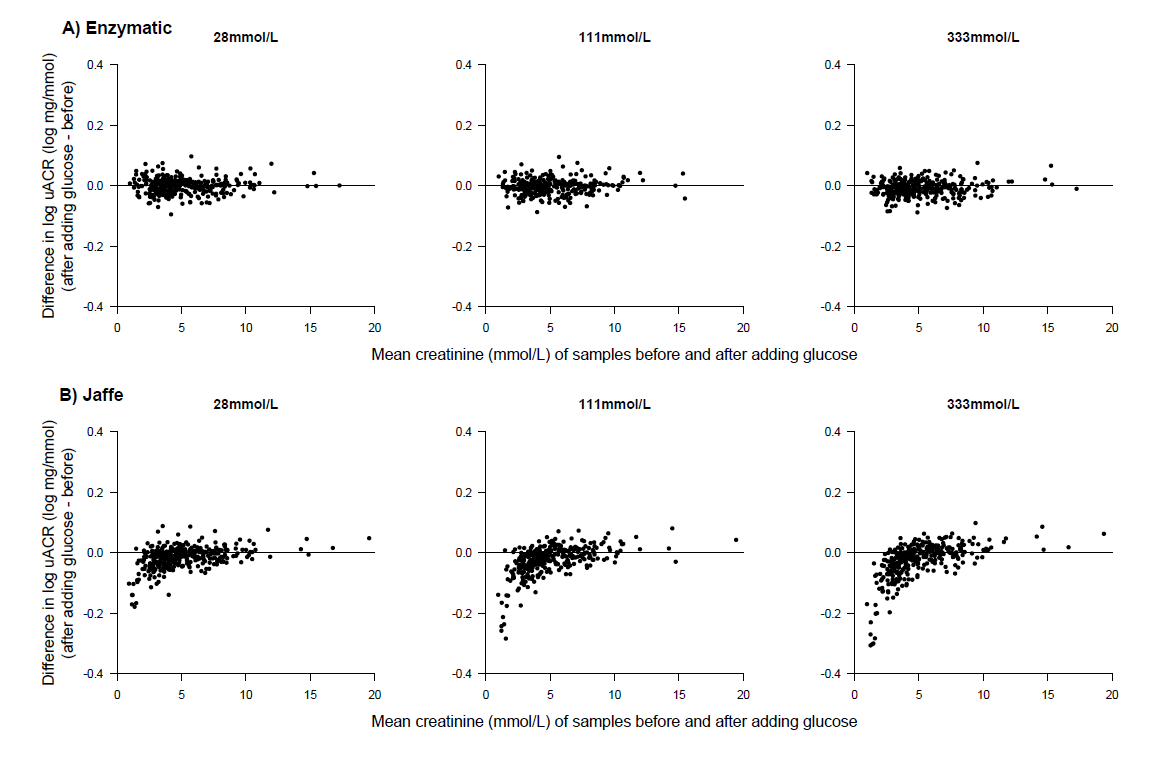


## Figure S4: Creatinine before and after adding glucose, by glucose concentration


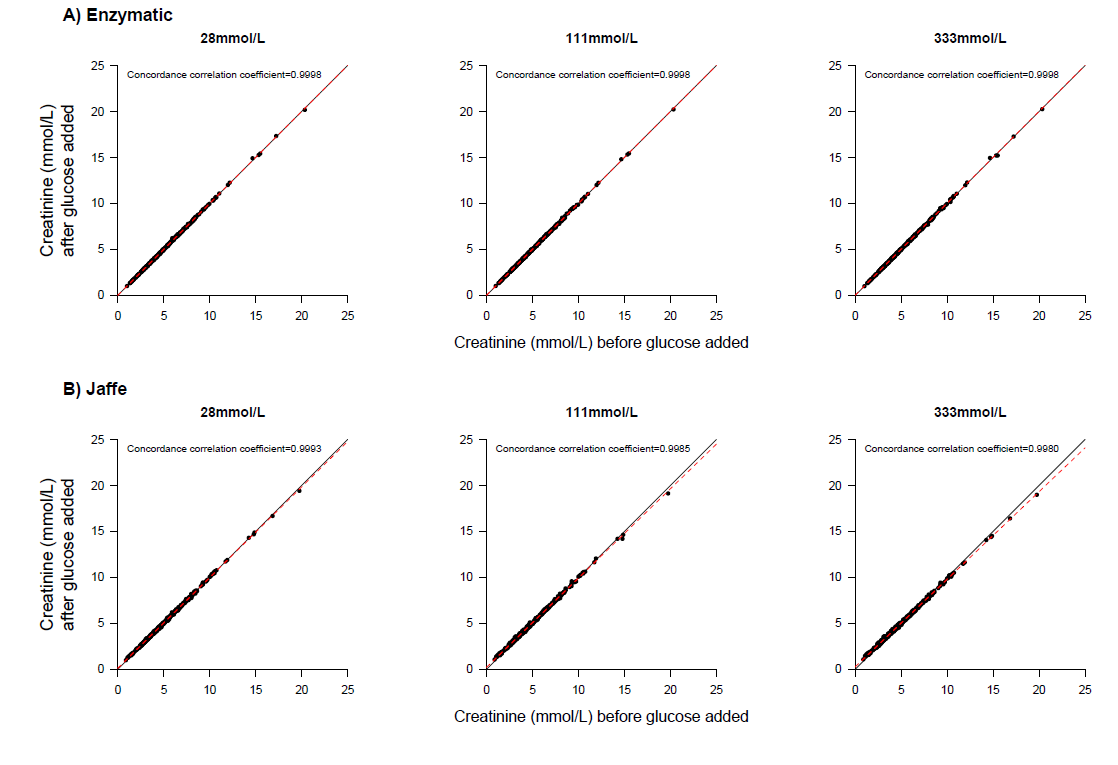


## Figure S5: uACR before and after adding glucose, by glucose concentration


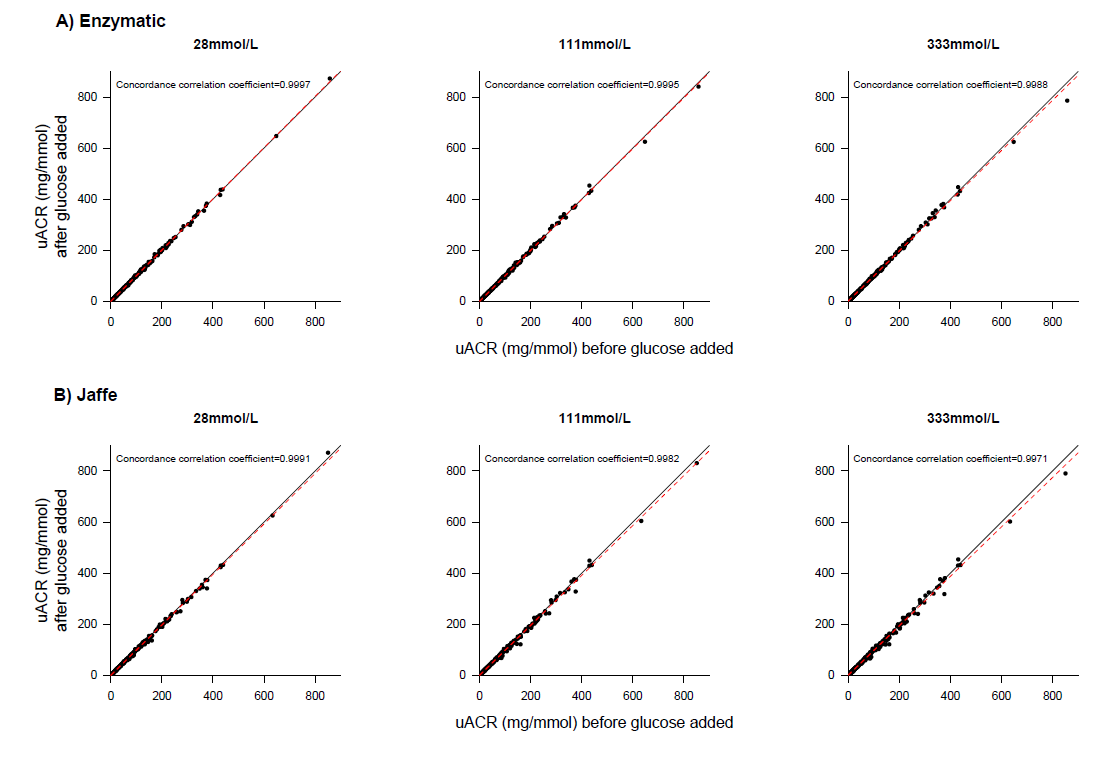


## Figure S6: Bland-Altman plots for uACR (untransformed), by glucose concentration

## Figure S7: Bland-Altman plots for albumin (untransformed), by glucose concentration

# **Supplementary References**

S1. UK HARP-III Collaborative Group. Randomized multicentre pilot study of sacubitril/valsartan versus irbesartan in patients with chronic kidney disease: United Kingdom Heart and Renal Protection (HARP)- III-rationale, trial design and baseline data. *Nephrol, Dial, Transplantation.* 2017;32(12):2043-2051.

S2. Herrington W, Illingworth N, Staplin N, et al. Effect of Processing Delay and Storage Conditions on Urine Albumin-to-Creatinine Ratio. *Clin J Am Soc Nephrol.* 2016;11(10):1794-1801.

S3. Chapman DP, Gooding KM, McDonald TJ, et al. Stability of urinary albumin and creatinine after 12 months storage at -20 degrees C and -80 degrees C. *Pract Lab Med* 2019;15:e00120.

S4. Kim SR, Lee YH, Kang ES, et al. The Relationship between Increases in Morning Spot Urinary Glucose Excretion and Decreases in HbA(1C) in Patients with Type 2 Diabetes After Taking an SGLT2 Inhibitor: A Retrospective, Longitudinal Study. *Diabetes Ther.* 2017;8(3):601-609

S5. Seegmiller JC, Miller WG, Bachmann LM. Moving Toward Standardization of Urine Albumin Measurements. *EJIFCC.* 2017;28(4):258-267.

S6. Bland JM, Altman DG. Statistical methods for assessing agreement between two methods of clinical measurement. *Lancet.* 1986;1(8476):307-310.

S7. Bland JM, Altman DG. The use of transformation when comparing two means. *BMJ.* 1996;312(7039):1153.

S8. Bland JM, Altman DG. Transforming data. *BMJ.* 1996;312(7033):770.

S9. Wiviott SD, Raz I, Bonaca MP, et al. Dapagliflozin and Cardiovascular Outcomes in Type 2 Diabetes. *N Engl J Med.* 2019;380(4):347-357.

S10. Mosenzon O, Wiviott SD, Cahn A, et al. Effects of dapagliflozin on development and progression of kidney disease in patients with type 2 diabetes: an analysis from the DECLARE-TIMI 58 randomised trial. *Lancet Diabetes Endocrinol.* 2019;7(8):606-617.

S11. Perkovic V, Jardine MJ, Neal B, et al. Canagliflozin and Renal Outcomes in Type 2 Diabetes and Nephropathy. *N Engl J Med.* 2019;380(24):2295-2306.

S12. Perkovic V, de Zeeuw D, Mahaffey KW, et al. Canagliflozin and renal outcomes in type 2 diabetes: results from the CANVAS Program randomised clinical trials. *Lancet Diabetes Endocrinol.* 2018;6(9):691-704.

S13. Cannon CP, Pratley R, Dagogo-Jack S, et al. Cardiovascular Outcomes with Ertugliflozin in Type 2 Diabetes. *N Engl J Med.* 2020;383(15):1425-1435.

S14. Wanner C, Inzucchi SE, Lachin JM, et al. Empagliflozin and Progression of Kidney Disease in Type 2 Diabetes. *N Engl J Med.* 2016;375(4):323-334.

S15. Cherney DZI, Zinman B, Inzucchi SE, et al. Effects of empagliflozin on the urinary albumin-to-creatinine ratio in patients with type 2 diabetes and established cardiovascular disease: an exploratory analysis from the EMPA-REG OUTCOME randomised, placebo-controlled trial. *Lancet Diabetes Endocrinol.* 2017;5(8):610-621.

S16. McMurray JJV, Solomon SD, Inzucchi SE, et al. Dapagliflozin in Patients with Heart Failure and Reduced Ejection Fraction. *N Engl J Med.*2019;381(21):1995-2008.

S17. Packer M, Anker SD, Butler J, et al. Cardiovascular and Renal Outcomes with Empagliflozin in Heart Failure. *N Engl J Med.* 2020;383(15):1413-1424.

S18. Anker SD, Butler J, Filippatos G, et al. Empagliflozin in Heart Failure with a Preserved Ejection Fraction. *N Engl J Med.* 2021;385(16):1451-1461.

S19. Bhatt DL, Szarek M, Steg PG, et al. Sotagliflozin in Patients with Diabetes and Recent Worsening Heart Failure. *N Engl J Med.* 2020;384(2):117-128.

S20. Bhatt DL, Szarek M, Pitt B, et al. Sotagliflozin in Patients with Diabetes and Chronic Kidney Disease. *N Engl J Med.* 2020;384(2):129-139.

S21. Heerspink HJL, Stefánsson BV, Correa-Rotter R, et al. Dapagliflozin in Patients with Chronic Kidney Disease. *N Engl J Med.* 2020;383(15):1436-1446.

S22. ACCORD Study Group, Cushman WC, Evans GW, et al. Effects of intensive blood-pressure control in type 2 diabetes mellitus. *N Engl J Med.* 2010;362(17):1575-1585.

S23. UK Prospective Diabetes Study Group. Tight blood pressure control and risk of macrovascular and microvascular complications in type 2 diabetes: UKPDS 38. UK Prospective Diabetes Study Group. *BMJ.* 1998; 317(7160): 703-713.

24. UK Prospective Diabetes Study Group. UK Prospective Diabetes Study (UKPDS). VIII. Study design, progress and performance. *Diabetologia.* 1991; 34(12): 877-890.

S25. The ADVANCE Collaborative Group. Intensive Blood Glucose Control and Vascular Outcomes in Patients with Type 2 Diabetes. *N Engl J Med.*2008;358(24):2560-2572.

S26. Duckworth W, Abraira C, Moritz T, et al. Glucose Control and Vascular Complications in Veterans with Type 2 Diabetes. *N Engl J Med.* 2009;360(2):129-139.
